# Disparities in lipid management for African Americans and Caucasians with coronary artery disease: A national cross-sectional study

**DOI:** 10.1186/1471-2261-4-15

**Published:** 2004-08-18

**Authors:** Mark W Massing, Kathleen A Foley, Lori Carter-Edwards, Carla A Sueta, Charles M Alexander, Ross J Simpson

**Affiliations:** 1 Health Care Assessment, Medical Review of North Carolina, Cary, North Carolina, USA; 2 Departmment of Epidemiology, School of Public Health, University of North Carolina, Chapel Hill, North Carolina, USA; 3 Outcomes Research and Management, Merck & Company, West Point, Pennsylvania, USA; 4 Institute for Health, Social, and Community Research, Shaw University, Raleigh, North Carolina, USA; 5 Department of Cardiology, School of Medicine, University of North Carolina, Chapel Hill, North Carolina, USA

## Abstract

**Background:**

Individuals with coronary artery disease are at high risk for adverse health outcomes. This risk can be diminished by aggressive lipid management, but adherence to lipid management guidelines is far from ideal and substantial racial disparities in care have been reported. Lipid treatment and goal attainment information is not readily available for large patient populations seen in the fee-for-service setting. As a result, national programs to improve lipid management in this setting may focus on lipid testing as an indicator of lipid management. We describe the detection, treatment, and control of dyslipdemia for African Americans and Caucasians with coronary artery disease to evaluate whether public health programs focusing on lipid testing can eliminate racial disparities in lipid management.

**Methods:**

Physicians and medical practices with high numbers of prescriptions for coronary artery disease medications were invited to participate in the Quality Assurance Program. Medical records were reviewed from a random sample of patients with coronary artery disease seen from 1995 through 1998. Data related to the detection, treatment, and control of dyslipidemia were abstracted from the medical record and evaluated in cross-sectional stratified and logistic regression analyses using generalized estimation equations.

**Results:**

Data from the medical records of 1,046 African Americans and 22,077 Caucasians seen in outpatient medical practices in 23 states were analyzed. African-American patients were younger, more likely to be women and to have diabetes, heart failure, and hypertension. The low density lipoprotein cholesterol (LDL-C) testing rate for Caucasian men was over 1.4 times higher than that for African-American women and about 1.3 times higher than that for African-American men. Almost 60% of tested Caucasian men and less than half of tested African Americans were prescribed lipid-lowering drugs. Tested and treated Caucasian men had the highest LDL-C goal attainment (35%) and African-American men the lowest (21%).

**Conclusions:**

Although increased lipid testing is clearly needed for African Americans, improvements in treatment and control are also necessary to eliminate racial disparities in lipid management. Disparities in treatment and goal attainment must be better understood and reflected in policy to improve the health of underserved populations.

## Background

Individuals with coronary disease (CAD) are at high risk for subsequent cardiovascular disease events and mortality[1]. Clinical trials have shown that this risk can be substantially reduced though the detection, treatment, and control of dyslipidemia[2,3]. To that end, clinical guidelines have been established for the lipid management of CAD patients[4,5]. Adherence to these guidelines is far from ideal[6,7]. Substantial racial disparities in the diagnosis and management of dyslipidemia have been reported in the general population and among CAD patients [8-16].

Improved lipid management through the diagnosis of dyslipidemia has been a focus of quality improvement programs in the outpatient fee-for-service setting such as Medicare's Health Care Quality Improvement Program[17]. Lipid testing is used as an indicator of lipid management in the fee-for-service setting because testing is a service identifiable in insurance claims data. In contrast, assessment of treatment and goal attainment in the fee-for-service setting requires resource-intensive medical record review which is generally not performed for large national patient populations.

This study describes outpatient lipid management for African Americans and Caucasians with CAD seen in medical practices throughout the United States. Data from medical records were examined for indicators of lipid management including lipid testing, lipid-lowering drug prescription, and goal attainment. Our objectives were to characterize lipid management across race-sex groups and evaluate the extent of disparities for the three components of lipid management: detection, treatment, and control. We then discuss the implications of our findings with respect to possible underlying causes and health policies for closing the gap in race-sex lipid management disparities.

## Methods

### The Quality Assurance Program

The Quality Assurance Program (QAP) is a national program sponsored by Merck & Company conducted during the late 1990's to identify physician practice patterns and to promote evidence-based best practices for the medical management of patients with cardiovascular disease seen in the outpatient setting [18]. The QAP database provides abstracted medical record data collected in two distinct time periods and study populations nationwide. Our analyses were limited to the study population identified in the most recent period of data collection (QAP-II). Patients from QAP-II included in these analyses were seen at participating medical practices from January, 1995 through March, 1998.

Medical records were reviewed by Access Medical Ltd (Arlington, VA) using a standardized electronic abstraction tool developed specifically for QAP-II. The QAP database contains data obtained from the medical record including race, sex, date of birth, medical history, and medical procedures. The most recent serum lipid testing results and the most recently recorded prescriptions for lipid-lowering drugs were also determined from the medical record. The medical record of each patient was reviewed only once. Patients were not followed over time. Patient and physician identifying information were not included in the QAP-II database to ensure confidentiality.

### QAP participant selection

Physicians and medical practices throughout the United States with high numbers of prescriptions for medications used in the treatment of cardiovascular disease were invited to participate in the QAP. The specialties of participating physicians included cardiology, internal medicine, family medicine, and endocrinology. Patients with cardiovascular disease were randomly selected within each participating medical practice.

### Inclusion and exclusion criteria

Patients included in the QAP study were at least 21 years of age with CAD and/or heart failure and were seen at least twice in two years by the participating physician. Patients were excluded if the medical record indicated a terminal illness, history of a transplant or awaiting transplant, or deceased.

Patients without medical record documentation of CAD were excluded from analysis. The presence of CAD was ascertained during analysis from abstracted medical record data based on medical history, *International Classification of Diseases, Ninth Revision, Clinical Modification *diagnosis codes (410–414), and cardiac procedures consistent with CAD (i.e., coronary artery bypass graft, angioplasty, and stent).

Only patients with medical record documentation of African-American or Caucasian race were included. To reduce the influence of between-state variation in lipid management from states that contribute little information about African American populations, medical practices were excluded if they were located in states with fewer than 10 African-American patients in QAP-II

### Indicators of lipid management

Measures of detection (lipid testing), treatment (lipid-lowering drug prescription), and control (goal attainment) were the indicators of lipid management considered in this study. Low density lipoprotein cholesterol (LDL-C) testing was measured as the percentage of patients with at least one serum LDL-C value documented in the medical record. The use of lipid-lowering drugs (i.e., "treated" patients) was measured as the percentage of patients with medical record documentation of at least one prescription for a statin (3-hydroxy-3-methylglutaryl coenzyme A reductase inhibitor) or non-statin lipid drug (e.g., gemfibrozil). Goal attainment among those with documented LDL-C values was based on recommended guidelines for patients with coronary artery disease (LDL-C < 100 mg/dL)[5].

### Analysis

We conducted a cross-sectional analysis of abstracted medical record data obtained from QAP-II. Indicators of lipid management (i.e., LDL-C testing, lipid-lowering drug prescription, and LDL-C goal attainment rates) and potential confounding or explanatory variables were examined within strata of race and sex. Co-morbid conditions including diabetes mellitus, myocardial infarction, heart failure, and hypertension were identified from medical history and diagnosis codes.

Logistic regression analyses were performed to evaluate the associations of race and sex with each of three dichotomous lipid management indicators while controlling for multiple confounding and explanatory variables. We accounted for correlations within medical practices using the generalized estimation equation (GEE) method[19]. This method was implemented in generalized linear models with PROC GENMOD of SAS Version 9 (SAS, Inc, Cary, North Carolina) for logistic regression with correlated data[20]. Separate models were run for each indicator of lipid management as the dependent variable. The entire study population was included for logistic regression analyses of LDL-C testing as the dependent variable. Regression analyses with lipid-lowering drug prescription as the dependent variable included only patients with LDL-C tests. Regression analyses for LDL-C goal attainment were limited to patients who received at least one LDL-C test and had a documented prescription for a lipid-lowering drug.

The independent variables included in the regression models in addition to race and sex were age, medical history (diabetes mellitus, myocardial infarction, heart failure, hypertension), and geographic region of medical practice. Logistic models predicting lipid-lowering drug prescriptions included a term for serum LDL-C concentration in addition to the above variables. LDL-C concentration was included to control for the severity of hyperlipidemia as a factor in the physician's decision to treat with lipid-lowering drugs.

## Results

### Population characteristics

A total of 23,123 CAD patients with documented race and sex seen by 1,171 physicians at 238 medical practices in 23 states were included in the study. Of these patients, 1,046 were African American and 22,077 were Caucasian. African-American compared to Caucasian patients were more likely to be women (53% versus 36%). The average age for the study population was 69 years and ranged from 22 to 97 years.

Within each race group, women were older than men on average and within each sex group Caucasians were older than African Americans (Table 1). The prevalence of co-morbid conditions was high for all race-sex groups. Despite their younger ages, African Americans were more likely than Caucasians to have diabetes, heart failure, and hypertension. Almost half of African-American women and a third of African-American men had diabetes compared to about a quarter of the Caucasian population. About half of African-American men had heart failure and over three quarters of African-American women had hypertension. Consistent with the geographic distribution of race-specific populations, African-American patients were more likely seen in southern medical practices than elsewhere.

**Table 1:** Characteristics of patients by race and sex.*

Characteristic	African Americans		Caucasians	
	Women (n = 558)	Men (n = 488)	Women (n = 8,038)	Men (n = 14,039)
Age (mean ± SEM)	66.8 ± 0.5	62.7 ± 0.6	72.1 ± 0.1	67.1 ± 0.1
Medical History				
Diabetes Mellitus	43	33	25	21
Myocardial Infarction	38	47	40	48
Heart Failure	42	48	39	34
Hypertension	78	70	63	52
Region				
Northeast	27	25	34	31
Midwest	24	30	31	33
South	42	36	22	23
West	8	8	13	13
Serum Lipid Levels				
LDL-C (mean ± SEM mg/dl)^†^	131.3 ± 2.5	133.0 ± 2.5	124.8 ± 0.6	117.2 ± 0.3
LDL-C at goal (%)^†^	19	18	25	31

*Percent of race- and sex-specific total unless otherwise specified. ^†^For patients with documented tests having valid values

### Lipid testing

**Figure 1:**
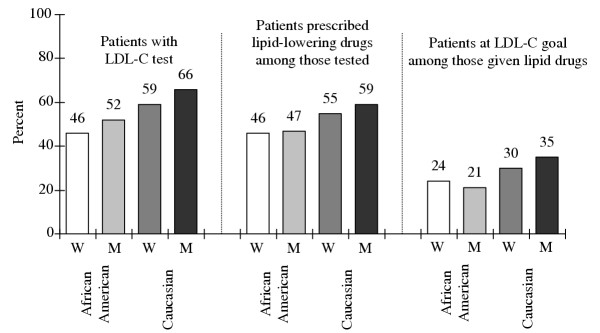
**Lipid Management among CAD patients. **Lipid testing, treatment, and goal attainment rates for African-American and Caucasian women (W) and men (M).

Within sex strata, the percent of patients with LDL-C tests was lower for African-Americans compared to Caucasians (Figure 1). Within race strata the percent of patients with LDL-C tests was lower for women than for men. The LDL-C testing rate for Caucasian men was over 1.4 times higher than that for African-American women and about 1.3 times higher than that for African-American men.

### Lipid treatment

Almost 60% of Caucasian men with LDL-C testing were prescribed lipid-lowering drugs (Figure 1). The proportion of tested Caucasian women receiving these drugs (55%) was similar to, but slightly lower than that for Caucasian men. Less than half of tested African-American men (47%) and women (46%) were prescribed lipid-lowering drugs.

### Goal attainment

Including those prescribed and not prescribed lipid-lowering drugs (Table 1), a quarter of Caucasian women and less than 20% of African-American men and women achieved the recommended LDL-C goal. But a higher proportion (31%) of Caucasian men achieved goal. The mean serum LDL-C concentration for Caucasian men (117 mg/dL) was lower than that for Caucasian women (125 mg/dL) and for African-Americans. Mean LDL-C concentrations were similar for African-American men (133 mg/dL) and women (131 mg/dL).

Among patients tested and treated with lipid-lowering drugs about two-thirds or more failed to achieve goal (Figure 1). Tested and treated Caucasian men had the best goal attainment rates (35%). Tested and treated Caucasian women had lower levels of goal attainment than Caucasian men. Goal attainment for Caucasian men and for women of either race exceeded that of African-American men. Only about 1 of 5 African-American men prescribed lipid-lowering drugs achieved LDL-C goal.

### Logistic regression

Results from GEE logistic regression analyses controlling for age, co-morbid conditions, and geographic region (Table 2) were consistent with results from the stratified analyses described above. Relative to Caucasians, African-American men and women were under-tested, under-treated, and less likely to be at goal. Relative to men, regardless of race, women were less likely to be tested. But if tested, women were as likely as men of the same race to receive prescriptions for lipid-lowering drugs. African-American women were the least likely to be tested (Odds Ratio, OR = 0.49), and if tested, African-American men were the least likely to be prescribed lipid drugs (OR = 0.59). Among those tested and treated, African-American men were least likely to be at goal (OR = 0.47). That is, among tested patients who were prescribed lipid-lowering drugs, the odds for goal attainment for Caucasian men were more than twice the odds for African-American men.

**Table 2:** Lipid testing, pharmacologic treatment, and goal attainment among African-American men and women and Caucasian women relative to Caucasian men from logistic regression analyses.*

	African American		Caucasian	
	Women Odds Ratio (95% CI)*	Men Odds Ratio (95% CI)*	Women Odds Ratio (95% CI)*	Men Reference Group
LDL-C tested (n = 23,104)	0.49 (0.37,0.64)	0.60 (0.47,0.77)	0.80 (0.74,0.86)	1.00
Lipid drug prescribed (n = 14,499)^†^	0.62 (0.46,0.83)	0.59 (0.45,0.78)	1.04 (0.95,1.13)	1.00
LDL-C goal attainment (n = 8,336) ^‡^	0.55 (0.36,0.82)	0.47 (0.30,0.74)	0.76 (0.68,0.85)	1.00

*Odds ratio and 95% confidence intervals (95% CI) from logistic regression models accounting for within-practice correlations with GEE and controlling for race, sex, age, medical history (diabetes mellitus, myocardial infarction, heart failure, hypertension), and geographic region of medical practice. ^†^Regression model includes serum LDL-C concentration. ^‡^LDL-C goal attainment (<100 mg/dL) among those with documented LDL-C values and treated with lipid-lowering drugs.

## Discussion

Consistent with previous reports, our findings demonstrate that outpatient lipid management for CAD patients in the late 1990's had much room for improvement and that substantial race-sex disparities existed[8,12,16,21-26]. African Americans experienced markedly lower levels of LDL-C testing than Caucasians and, as a result, they may benefit more than Caucasians from interventions to improve testing. Among those tested, African Americans were less likely to be treated and, if treated, they were less likely to be at goal compared to Caucasians. Marked lipid testing disparities by sex suggest a need for more aggressive testing in women.

Much of the information needed to assess lipid management can be found only in the medical record. This information is generally not available to national and local public health programs attempting to implement policies promoting quality improvement in the many and diverse medical practices treating large patient populations. The most readily available data for patients seen in the fee-for-service setting is derived from administrative insurance claims for the reimbursement of costs associated with drugs and services. Insurance coverage for lipid-lowering drugs varies across plans. In contrast, lipid testing for CAD patients is a widely covered service that can be identified using electronic billing data without the need to review patient records in medical practices. For this reason, lipid testing is a focus of national efforts in the Medicare population to improve outpatient lipid management in the fee-for-service setting[17]. Our findings demonstrate that substantial disparities in treatment and goal attainment exist among CAD patients with lipid tests. This implies that current public health programs and policies designed to increase lipid testing alone will have limited impact on lipid management disparities. Substantial disparities in lipid treatment and control will likely persist in the absence of disparities in testing.

Underlying causes of inadequate lipid management among CAD patients are multiple and likely vary by race and process of care (i.e., detection, treatment, and control). Factors that limit patient-physician encounters and continuity of care may partially account for racial disparities in lipid management. For example, African-American Medicare consumers with diabetes were more likely to receive outpatient care from emergency departments and had fewer physician visits per year than their Caucasian counterparts[12,27]. The QAP data provide insufficient information to evaluate whether health care access and continuity explain lipid management disparities.

A report regarding racial disparities in the use of prescription drugs from the Center for Studying Health System Change provides evidence of other factors explaining racial disparities in lipid management[11]. In this report, Medicare consumers 65 years of age and older were surveyed regarding their ability to obtain prescription drugs. African Americans were more than twice as likely as Caucasians to have not filled a prescription because they could not afford it. More than 16% of Medicare insured African Americans reported that they could not afford to fill at least one prescription in 2001. One-fifth of African Americans and 13% of Caucasians with low income could not afford to fill at least one prescription. During the time period of QAP-II, Medicare did not cover the cost of lipid-lowering drugs. Many Medicare consumers have supplemental insurance that assists with drug costs. In the Medicare population, African Americans were less likely than Caucasians to have supplemental insurance and more likely to be of low income[11].

Barriers to lipid management due to affordability may result in racial disparities if affordability differs by race. Patients who can not afford treatment may be less likely to aggressively pursue it with their physicians and may be less likely to comply with physician recommendations for testing and treatment. Secondary prevention of cardiovascular diseases among CAD patients with pharmacologic agents such as statins has been shown to be cost effective[28]. But drug costs that may exceed $2,000 annually can be beyond the reach of low income and underinsured patients[29]. These costs are more likely a barrier for African Americans than for Caucasians and may contribute to lipid management disparities.

The fact that African Americans are more likely to be of low income has greater implications than simply the inability to afford medications. Income is one of several indicators of socioeconomic status correlated with factors related to health including education[30]. Low education has been identified as a factor limiting a patient's personal involvement in lipid management[31]. There is evidence that African Americans are less knowledgeable about cholesterol compared to Caucasians[24]. African Americans may be less aware of the need for lipid management and less likely to pursue it with their physician.

Achieving LDL-C goal is challenging for all races, but African Americans may require especially aggressive lipid management accompanied by an enhanced understanding of reasons for failure to achieve goal. A recent report concerning patients with CAD and/or diabetes seen at a Veterans Affairs Medical Center found that African Americans were less likely to achieve lipid goal than Caucasians when prescribed identical doses of the same lipid-lowering drug even though African Americans had more clinic visits and lipid tests[10]. The authors speculate that racial disparities in goal attainment may have occurred due to differences in compliance, lifestyle, and baseline LDL-C (higher for African Americans). In a study of LDL-C lowering with pravastatin in an African-American population, only 13% of patients with a LDL-C goal of 100 mg/dL actually achieved it. Incorrect drug regimen, inadequate lipid monitoring, and compliance problems were thought to have contributed to these goal attainment failures[32]. Additional studies are needed to investigate the underlying causes of lower goal attainment for African Americans receiving treatment for dyslipidemia.

Physicians have indicated that oversight is a common reason for failure to adhere to lipid testing guidelines[33]. Whether oversight contributes to racial disparities in lipid management is not known, but oversight can be alleviated by system changes in the medical practice[34]. Awareness is growing that implementation of electronic health records is a necessary component of efforts to improve healthcare quality and prevent medical errors[35,36]. The impact of electronic systems on disparities is an interesting area for future research. Lipid management is also influenced by physician attitudes about guidelines and drug effectiveness[37]. A better understanding is needed of physician attitudes and their relations with healthcare disparities.

Physician-patient interactions are influenced by race and cultural factors related to race. African-American and Caucasian patients may differ with respect to cultural perceptions of health and disease and the ability of patients to influence or control their health outcomes[38]. African Americans may be less likely than Caucasians to trust their physicians and racial groups may view their relationship with physicians differently[39]. A survey of adults seen in a managed care setting revealed that African Americans viewed their visits with physicians as less participatory than did Caucasians, but they felt more participatory when seeing a physician of their own race[40].

The relatively poorer lipid management for African Americans compared to Caucasians may be partially explained by racial differences in the prevalence of co-morbid conditions. African Americans in the QAP were more likely than Caucasians to suffer from multiple chronic conditions including diabetes and heart failure. It has been shown that patients with diabetes compared to those without diabetes receive poorer lipid management and are less likely to be at goal[41]. An earlier report from the QAP has shown that patients with CAD and heart failure were less likely to receive lipid testing and cholesterol-lowering drugs than those without heart failure[42]. Co-morbid conditions may hinder lipid management in a number ways. These patients may be more likely to have contra-indications to lipid treatment and to present with acute life-threatening conditions that divert attention away from lipid management. In addition, it has been reported that patients with multiple chronic conditions are less likely to afford medications than those with one or fewer chronic conditions[11]. But even after controlling for several highly prevalent co-morbid conditions in logistic regression analyses, we find that significant lipid management disparities persist.

There are a number of limitations in the QAP study design and its population that potentially pose a threat to internal and external validity. These limitations suggest that our findings may not reflect the experiences of the general population. The medical practices included in the QAP were restricted to those writing large numbers of prescriptions for cardiovascular disease drugs. It is difficult to evaluate the impact of this selection bias, but we suspect that medical practices included in the QAP represent the larger and more sophisticated providers of care. Thus, lipid management among QAP participants may be better than that found in the general population. Another limitation to our study is the lack of information characterizing medical practices, physicians, and patients with respect to factors related to race, sex, and lipid management. Because of the unavailability of this information, we could not identify underlying factors potentially explaining our findings. Lipid management data for the QAP patients seeing multiple physicians were unavailable from physicians not participating in QAP and this may have influenced results in an unpredictable manner.

Considering the relatively small proportion of the study population identified as African American (<5%), it is likely that African Americans in this study do not represent the national population. We have no specific information about QAP medical practices or their patient populations, but we speculate that African Americans in the QAP were likely receiving better care on average than their counterparts in the general population, especially those with little or no access to outpatient care. If this is true, then racial disparities in lipid management in the general population may be even greater that those suggested by our findings.

The time frame for this study includes years 1995 through 1998. Despite changes in guidelines and therapies since that time, our findings remain relevant to current practices. They provide historic context and baseline measures of lipid management disparities necessary to evaluate trends. In addition, they direct attention to the persistent need to provide aggressive treatment to high risk populations with CAD, especially African Americans and women who continue to be underserved. Finally, they highlight the growing view that public health strategies in lipid management must shift focus from testing to treatment and goal attainment[43].

Clearly, a multi-pronged approach including all three elements of the process of care (i.e., detection, treatment, and control) is needed to improve lipid management for all race-sex groups, and particularly for African Americans. Although beyond the scope of this report, the application of conceptual models to the process of care may be useful in understanding how racial disparities arise at each step of the process. Furthermore, policies addressing health promotion and non-medical determinants of health such as socioeconomic status, community environment, and lifestyle choices need also be considered in confronting these disparities[44,45].

Successful lipid management likely depends on a variety of processes that determine the provision of medical services including their availability, accessibility, and acceptability. The substantial racial disparities in lipid management among patients in contact with medical providers suggest that the effectiveness of medical services and patient characteristics play a prominent role in lipid management. Policies promoting appropriate delivery of care through system change as well as those ensuring equal access to care are required to eliminate lipid management disparities in the population of high-risk CAD patients[34,46].

## Conclusions

Our results suggest that policies and programs focusing solely on the elimination of lipid testing disparities can have only limited benefit in reducing the major disparity in lipid management. The elimination of lipid management disparities will require policies that view untested, untreated, and under-treated individuals as separate populations with unique challenges and solutions[43].

Disparities in testing are just one element in explaining overall disparities in lipid management and ultimately, in cardiovascular outcomes. Future research should address patient, physician, and health system factors that lead to lower rates of testing, treatment and goal attainment for African Americans. Disparities in treatment and goal attainment must be better understood and reflected in policy in order to improve the health of underserved populations through optimal lipid management.

## List of abbreviations

CAD Coronary Artery Disease

LDL-C Low-density lipoprotein cholesterol

QAP Quality Assurance Program

GEE Generalized Estimation Equation

## Competing interests

Analyses were funded by an unrestricted grant from Merck & Co., Inc. No other competing interests are declared.

## Authors' contributions

MM conceived of the study, provided analytic and statistical support, and was the leading contributing author. KF participated in the design of the study and provided analytic support and interpretation of findings. LCE provided critical technical review and major contributions to the discussion section. CS provided conceptual guidance, assistance with QAP project database, and interpretation of analytic findings. CA provided critical review, QAP project experience, and contributions to the presentation and interpretation of findings. RS provided critical review, QAP project experience, and contributions to discussion and interpretation of findings. All authors read and approved the final manuscript.

## Pre-publication history

The pre-publication history for this paper can be accessed here:


